# Thermodynamic stability of magnetic states of monovacancy in graphene revealed by *ab initio* molecular dynamics simulations

**DOI:** 10.1038/s41598-018-37333-9

**Published:** 2019-01-24

**Authors:** Fei Gao, Shiwu Gao

**Affiliations:** Beijing Computational Science Research Center, 100193 Beijing, China

## Abstract

The stability of magnetic states is essential for potential spintronic applications. Here we report on the thermal stability of magnetic states of monovacancy graphene using *ab initio* molecular dynamics simulations. At room temperature, thermal fluctuations of the graphene lattice induce a rapid magnetic switching between two states with a high and low magnetic moment, indicating that due to the instability of the atomic structure of the vacancy, the associated magnetic moment is thermodynamically unstable. Lowering the temperature can significantly reduce the rate of the switching process and enhance the resident time on the high magnetic state. It stabilizes in the high magnetic state at as low as 30 K. Analyzing the atomic trajectories and the instant electronic structures confirms that these two magnetic states in MD simulations correspond to the magnetic and nonmagnetic states reported in the literatures. Such fluctuations of local magnetic moments are associated with the vertical displacement of the carbon atoms with the unsaturated dangling bond. This study reveals the dynamical correlation between atomic movement and the magnetic switching, and a comprehensive picture of vacancy magnetism in graphene. It has implications in graphene based spintronic devices.

## Introduction

Vacancy induced magnetism in graphene has attracted great attention in recent years due to its potential applications in spintronics and the fundamental interests in magnetic manipulations at nanoscale. While the pristine graphene is nonmagnetic, vacancies are known to carry a magnetic moment and have been intensively investigated over the past few years^1–10^. This magnetic moment originates from the removal of a single carbon atom, which breaks three σ dangling bonds in the graphene plane and a π bond in the perpendicular direction. Two of the σ bonds are saturated due to the Jahn-Teller effect^11,12^. The remaining σ dangling bond and the vacancy π band around the Fermi level gives rise to a local magnetic moment of 2 µ_B_. Because of the antiferromagnetic coupling between the vacancy states through the itinerant π bands, the magnetic moments are less than 2 µ_B_, usually 1.0 ~ 1.7 μ_B_ depending on the vacancy concentration^13–18^.

Although there seems to be a consensus on a magnetic ground state for graphene with a single vacancy, early reports in the literatures were controversial regarding a possible nonmagnetic ground state^17–19^. This nonmagnetic state has almost degenerate energy with the magnetic one but differs slightly in the geometric structure. For example, P. O. Lehtinen *et al*. pointed out that the nonmagnetic state was 0.1 eV higher in energy than the magnetic state, and the displacement of the dangling bond was 0.46 Å out of the graphene plane^18,19^. This conclusion was also supported by B. R. K. Nanda and coauthors, who obtained a flat structure with the magnetic moment of 1.5 µ_B_ and a deformed structure with a magnetic moment of 0.06 µ_B_. The two states differ in energy by about 50 meV^20^. In addition, a Kondo-like state was suggested by C. C. Lee *et al*. as the ground state^21^. The inconsistency in the ground state suggests that the spin polarization of monovacancy graphene might be thermodynamically unstable and thus casts doubt on its potential applications. So far, the thermal stability of defect graphene has not been investigated.

Here we perform *ab initio* molecular dynamics (AIMD) to study the thermodynamic stability of a single-atom vacancy in graphene and its correlation with magnetic properties at different temperatures. We found that magnetic states of monovacancy in graphene at room temperature is unstable, and it jumps between two states with a high and low magnetic moment. These magnetic switches are associated with the thermal fluctuations of the vertical coordinate (*h*) of the unsaturated carbon atoms close to the vacancy. At lowered temperatures such as 200 and 77 K, the rate of the magnetic switching can be substantially reduced and can be suppressed for a long time at 30 K, where a relatively stable ground state can be reached. Our study reveals the atomistic mechanism of thermal stability of monovacancy magnetism in graphene and has implications to graphene based spintronic devices.

## Results

**Figure 1:**
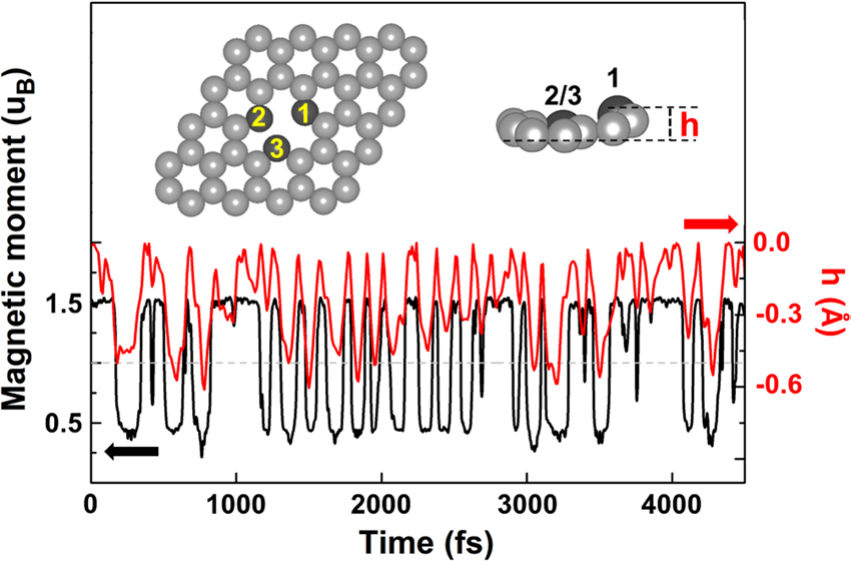
The time-dependent magnetic moment of monovacancy in the 6 × 6 graphene and the vertical displacement (*h*) between the atom 1 and the graphene plane at 300 K. Black and red solid lines represent the magnetic moment and the displacement. The insert shows the atomic geometry of the calculated supercell.

Figure 1 shows the time-dependent magnetic moment of monovacancy graphene at 300 K calculated by the 6 × 6 supercell. It is obvious that the rapid magnetic switching, at a time scale of 100 fs, occurs between two states with a high (1.5 µ_B_) and low (0.5 µ_B_) magnetic moment. Analysis of the atomic trajectories indicates that this magnetic switching is directly correlated with the local geometry fluctuations of the carbon atoms close to the vacancy, although vibration of the whole lattice is observable at this temperature (movie S1 in Supplementary Information, SI). As known before, two of the carbon atoms nearest to the vacancy (atoms 2 and 3 as labeled in the insert of Fig. 1) are much closer and re-bond due to the Jahn-Teller distortion, which saturates two dangling σ bonds and leaves the third atom, atom 1 here, an unsaturated σ bond possessing a local magnetic moment of 1 µ_B_. Interestingly, the fluctuation of the vertical displacement (*h*) between atom 1 and graphene plane leads to the magnetic switching directly (red solid line in Fig. 1). At *h* ≈ 0 Å, the magnetic moment is basically 1.5 µ_B_, whereas the magnetic moment jumps to 0.5 µ_B_ when *h* is larger than 0.3 Å in the 6 × 6 graphene. In contrast, the bond lengths between atoms 1 and 2 and between atoms 2 and 3 are also analyzed, which show no correlations with the magnetic transitions as shown in Fig. S1 in SI. The trajectories at room temperature indicate that the magnetic states of monovacany graphene are thermally unstable, and the instability is directly due to the vertical atomic deformation of the unsaturated carbon atom near the vacancy.

**Figure 2:**
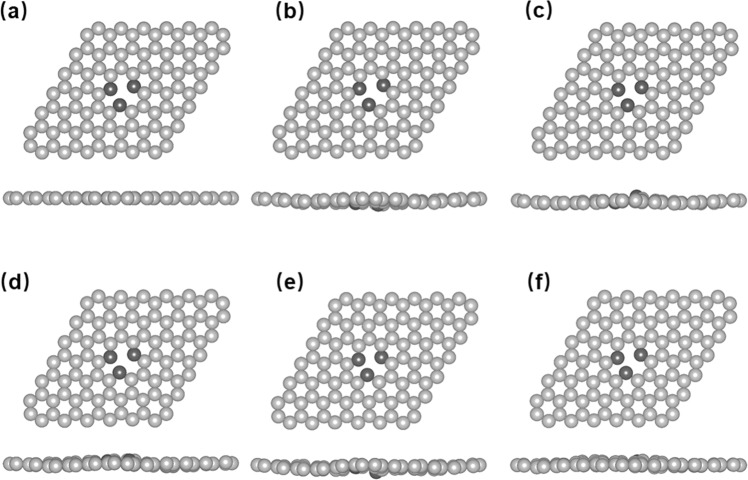
Snapshots of monovacancy in the 6 × 6 graphene at 300 K recorded at (**a**) t = 0 fs; (**b**) t = 500 fs; (**c**) t = 1500 fs; (**d**) t = 2500 fs; (**e**) t = 3500 fs; (**f**) t = 4500 fs, corresponding to the magnetic moment of 1.52, 1.32, 0.44, 1.50, 0.40, 1.39 µ_B_, respectively.

Figure 2 shows the snapshots of the lattice geometries recorded at *t* = 0, 500,1500, 2500, 3500, and 4500 fs, which correspond to the magnetic moment of 1.52, 1.32, 0.44, 1.50, 0.40, and1.39 µ_B_, respectively. Although the system jumps between the high and low magnetic states, the planar geometries display little variations, as shown by the top views of the snapshots. The in-plane stiffness results from the strong σ bonding within the graphene sheet. In the vertical direction, the carbon atoms vibrate up and down due to thermal excitation of the vibrational modes, and the vertical displacement of atom 1 has *h* = 0, 0.17, 0.60, 0.14, 0.56, and 0.14 Å, respectively. Therefore, it is clear that the magnetic transition directly results from the vertical fluctuation of the neighboring carbon atom. Moreover, the bond lengths between atom 1 and 2/3 are always larger than that between atom 2 and 3 during our 4.5 ps simulations as shown in Fig. S1 of SI. This does not exclude the possibility that the three atoms may interchange their positions and characters in longer times.

**Figure 3:**
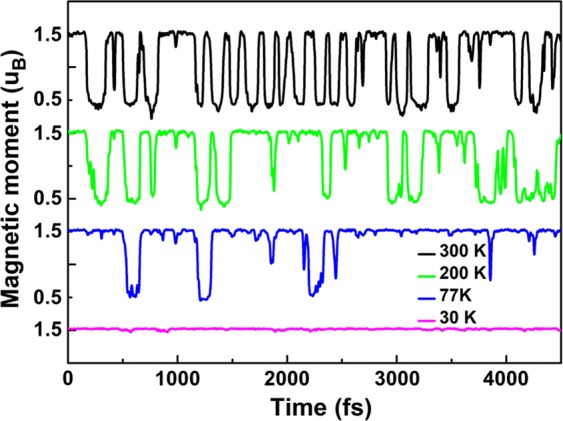
The time-dependent magnetic moment of monovacancy in the 6 × 6 graphene at 300 K (black solid line), 200 K (green solid line), 77 K (blue solid line) and 30 K (purple solid line).

Lowering the temperature to 200 and 77 K, the thermal fluctuations are suppressed, yet the switching between two magnetic states still persists at a lower rate. As shown in Fig. 3, the number of the magnetic jumps is much reduced in the 4.5 ps simulations and the resident time on the high magnetic moment (green and blue solid lines in Fig. 3) are increased, suggesting that the high magnetic state is the ground state (*S*_*g*_) and the low magnetic state is a metastable state (*S*_*m*_) with a higher energy. Only at a low temperature of 30 K, the magnetic switching in monovacancy graphene is suppressed state in the 4.5 ps simulations and the system is stabilized on the high magnetic ground. At the same time, the vibration of the carbon atoms is much damped, and the vertical displacement *h* stays smaller than 0.3 Å as shown in movie S2 of SI. Figure 3 suggests that lowering temperature is the robust way to achieve stability of the vacancy states. This is in line with the observation that experimental probe at liquid helium temperature of the defect magnetic state is indeed stable.

**Figure 4:**
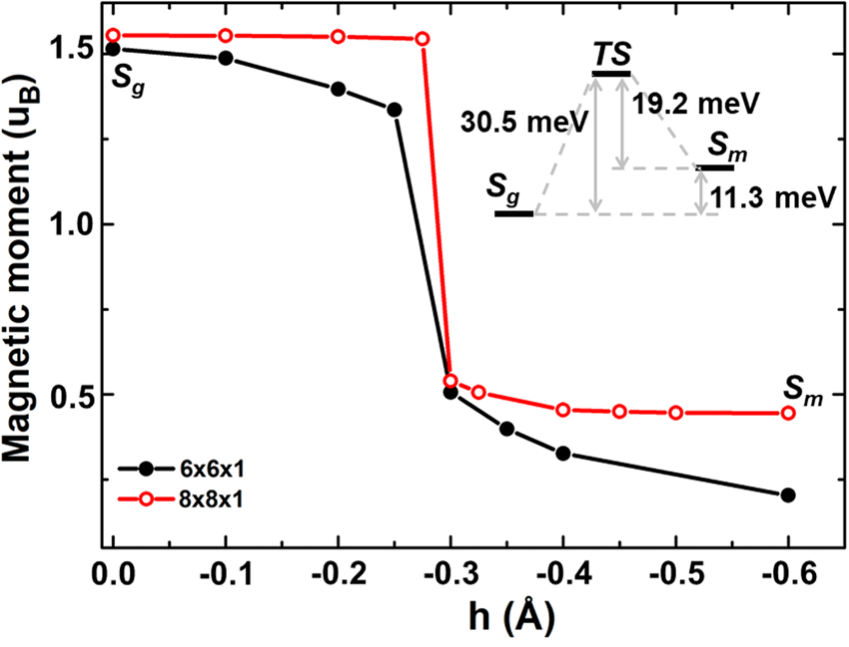
The magnetic moment as functions of the vertical displacement (*h*) between the atom 1 and graphene plane for the 6 × 6 and 8 × 8 graphene with single atom vacancy. The insert shows the chemical barrier and energy difference between the two states, *S*_*g*_ and *S*_*m*_.

The energy barrier between *S*_*g*_ and *S*_*m*_ can be deduced from the statistical residence time on the two states and the Arrhenius law for the transition rate across a barrier. The residence times on the two states at different temperatures are tabulated in Table 1. From the average residence time on one state at two temperatures, *T*_1_ and *T*_2_, the potential barrier (*E*_*b*_) can be deduced by,

1 $${E}_{b}={k}_{B}ln(\frac{{t}_{2}}{{t}_{1}})/(\frac{1}{{T}_{2}}-\frac{1}{{T}_{1}})$$

where *k*_*B*_ is the Boltzmann Constant, *t*_1_ and *t*_2_ are the average residence time on the same state at temperature *T*_1_ and *T*_2_, respectively. Using the average residence time on the ground state *t*_1_ = 98 fs and *t*_2_ = 177 fs at *T*_1_ = 300 and *T*_2_ = 200 K, the energy barrier from *S*_*g*_ to *S*_*m*_ is found to be 30.5 meV. Similarly, the energy barrier for the reverse process from *S*_*m*_ to *S*_*g*_, is 19.2 meV, with *t*′_1_ = 80 fs and *t*′_2_ = 116 fs on the metastable state at *T*_1_ = 300 and *T*_2_ = 200 K, respectively. From these two barriers, the energy difference between state 1 and 2 can be determined, which is 11.3 meV as schematically shown in the insert of Fig. 4. The average residence times on the two states at 77 K are also listed in Table 1, but it is statistically less reliable due to the few transitions in the 4.5 ps simulations.

**Table 1:** Average residence time on the ground state (*t*) and the metastable state (*t*′) at different temperatures (*T*).

*T* (K)	*t* (fs)	*t*′ (fs)
300	98	80
200	177	116
77	674	91

To further reveal the correlation between the local structures and the magnetic switching, we have done a series of static calculations by suspending the vertical displacement *h* at different heights while fully relaxing all other atomic coordinates in the 6 × 6 and the 8 × 8 graphene supercell. Figure 4 shows the calculated magnetic moment as a function of *h*. Clearly, a jump of the magnetic moment occurs at *h* = 0.3 Å for both set of calculations. Although the value of the magnetic moments changes slightly with the supercell, it confirms that the transition between the high and low magnetic states is present and occurs at about *h* = 0.3 Å. This conclusion is consistent with the results of the AIMD simulations. In addition, the small difference of magnetic moments is due to the sizes of the two supercells, which is in agreement with the previous works^13–18^.

The origin of the magnetic switching results from the inversion symmetry breaking in the monovacancy graphene, which undergoes a C_2v_ → C_2_ symmetry transformation in association with the vertical deformation around the vacancy defect. The C_2v_ symmetry of the magnetic ground state implies that the wave functions of the σ and the π vacancy states are orthogonal in space and do not hybridize. The two states can thus attain parallel spins according Hund’s rule of maximum multiplicity of spin occupation for independent orbitals, giving rise to the high magnetic state. In practice, the unsaturated σ state contributes to 1 µ_B_ as shown in Fig. S2 (a) of the SI. The partial occupancy of the π bands close to the Fermi level contributes less than 1 µ_B_ due to the antiferromagnetic coupling between vacancies at finite concentration. When atoms near the vacancy are deformed out of the plane by either thermal fluctuation or shear distortion^22^, the σ and π vacancy bands are no longer orthogonal and become hybridized, which can be clearly seen in their energy dispersion as shown in Fig. S2 (b) of the SI. The mixing between σ and π band due to inversion symmetry breaking changes therefore the occupation of the two states, leading to the reversal of spin polarization of the π bands near the Fermi level. The inversion symmetry breaking is therefore responsible for the magnetic switching and thermal instability. It also suggests that symmetry conservation, by any possible means like for example adsorption on substrate, can provide potential ways to stabilize the magnetic states of the defect graphene.

## Discussion

We present an *ab initio* MD study on the thermodynamic stability of a single-atom vacancy in graphene. At room temperature, the magnetic states of monovacancy graphene is thermally unstable. This instability originates from the inversion symmetry breaking associated with local atomic fluctuations perpendicular to the graphene plane. It is found that lowering temperature to 30 K can stabilize the system in the high magnetic state. This theoretical study provides dynamic insight into thermal stability of the magnetic states of monovacancy graphene. It also hints possible ways to control the graphene magnetism by symmetry conservation and thus has implications in spintronic applications.

## Methods

Our *ab initio* MD calculations are performed using the Vienna *ab initio* Simulation Package (VASP)^23^ based on density functional theory. It employs the projector augmented-wave (PAW)^24,25^ pseudopotentials and the general gradient approximation (GGA)^26^ in Perdew-Burke-Ernzerholf form^27^ for exchange-correlation energy. Spin polarization is included in all calculations. An energy cutoff of 600 eV is used for wave function expansion. The periodic supercells contain a vacuum layer of ~ 20 Å and all atoms are allowed to relax until the forces on each atom are less than 0.01 eV/Å. In the ground state, the C-C bond length is obtained to be 1.424 Å for the pristine graphene and is in agreement with the experimental value of 1.42 Å. The 6 × 6 supercell of graphene is used in the MD simulations with a time step of 1 fs and 3 × 3 × 1 k-sampling. The geometrical structure, electronic and magnetic properties of the MD snapshots of the single vacancy in graphene are compared with static 6 × 6 and 8 × 8 supercells with a finer grid of k-point sampling to check and assure the convergence of the simulations.

## Supplementary information

Supplementary Information

Supplementary Movie 1

Supplementary Movie 2
