# Supplementary material for: Thermodynamic stability of magnetic states of monovacancy in graphene revealed by ab initio molecular dynamics simulations
Source: Sci Rep. 2019 Jan 24;9:751. doi: 10.1038/s41598-018-37333-9 (PMC6345904; doi:10.1038/s41598-018-37333-9)
Supplement: Supplementary file 1 — Supplementary Information [file 41598_2018_37333_MOESM1_ESM.pdf]

Supplementary Information: Thermodynamic stability of magnetic states of monovacancy in graphene revealed by *ab initio* molecular dynamics simulations

Fei Gao and Shiwu Gao
Beijing Computational Science Research Center, 100193, Beijing, China

Supplementary Figures

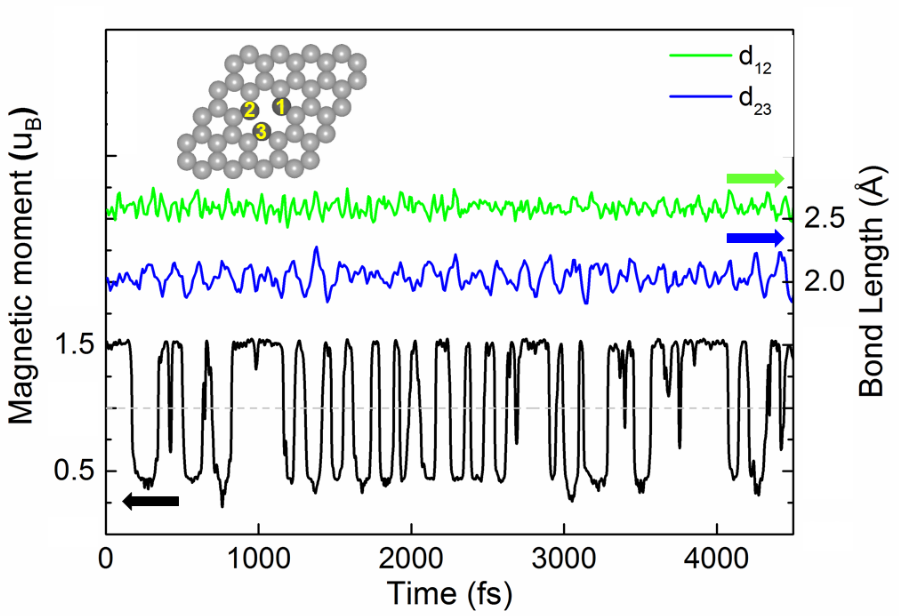

S1. The time-dependent magnetic moment (black solid line) of monovacancy in the 6 × 6 graphene and the bond lengths between atoms 1 and 2 (d_{12}) and between atoms 2 and 3 (d_{23}) at 300 K. Unlike the vertical displacement (h) in the main text, both bonds show no correlation with the magnetic switches. The insert shows the atomic geometry of the calculated supercell.

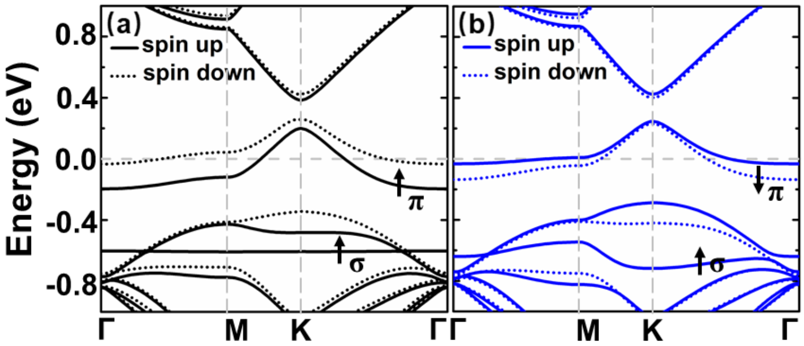

S2. Band structure of monovacancy in the 8 × 8 graphene for (a) ground state and (b) metastable state. Upon the vertical deformation, the localized σ band hybridizes with the π band of the vacancy state due to inversion symmetry breaking, which results in the magnetic switches.
